# Supplementary material for: Metabolic landscape of inborn errors of metabolism
Source: medRxiv. 2026 Oct 29:2026.10.28.26366400. Preprint. [Version 1] doi: 10.64898/2026.10.28.26366400 (PMC13734160; doi:10.64898/2026.10.28.26366400)

medRxiv preprint doi: https://doi.org/10.64898/2026.10.28.26366400; this version posted October 29, 2026. The copyright holder for this preprint (which was not certified by peer review) is the author/funder, who has granted medRxiv a license to display the preprint in perpetuity.
It is made available under a CC-BY 4.0 International license .

Supplementary Figure Legends

Figure S1. Related to Figure 1. Overview of IEM cohort.

A. Demographics of study subjects.

B. Summary of familial relationships in two geographic cohorts.

C. Top-ranked age-related metabolites in plasma. For all displayed metabolites, $p < 2x10^{-20}$ for correlation to age.

D. Top 20 most frequent metabolite outliers among all subjects.

E. Top 20 most frequent metabolite outliers among subjects with diagnosed IEMs.

Figure S2. Related to Figure 2. Summary of subjects with or without IEM diagnoses.

A. Demographics of patients diagnosed with known IEMs or without diagnoses. F, female; M, male.

B. Summary of the patients with deficiencies in different metabolic pathways or without diagnoses.

C. Levels of four relevant metabolites in patients with various *MMACHC* genotypes. ***p < 0.001, calculated by unpaired two-tailed t test.

Figure S3. Related to Figure 3. Metabolites altered in the plasma of patients with disorders of branched-chain amino acid oxidation.

A. The top-ranked diseases with differential levels of L-Carnitine.

B. Z-score distribution of C3-Carnitine abundance in all study subjects.

medRxiv preprint doi: https://doi.org/10.64898/2026.10.28.26366400; this version posted October 29, 2026. The copyright holder for this preprint (which was not certified by peer review) is the author/funder, who has granted medRxiv a license to display the preprint in perpetuity.
It is made available under a CC-BY 4.0 International license .

C. The top and bottom 25 outlier metabolites are shown for Maple Syrup Urine Disease (MSUD). The colored dots are plasma samples from subjects with MSUD. The GMDP subject number is shown for each of the five MSUD patients below the graph.

Figure S4. Related to Figure 4. Metabolomics in patient-derived fibroblasts to identify disease-associated metabolic alterations.

A. PCA plot showing unsupervised clustering of metabolomic profiles from fibroblasts obtained from healthy individuals (“unaffected”), and individuals with various diseases. “Diagnosed” and “mito diseases” indicate that the subject was diagnosed with an IEM. “Undiagnosed” indicates that the patient is being evaluated for an IEM, but no diagnosis has been established.

B. Box plots showing the abundance distributions of several outlier metabolites associated with GA1 in this cohort.

Figure S5. Related to Figure 5. Metabolomics and gene expression analyses in NADK2-deficient fibroblasts.

A. Pathway enrichment analysis of metabolites associated with NADK2 deficiency in the patient’s fibroblasts compared to control fibroblasts.

B. Immunoblotting of NADK2 protein in the patient’s fibroblasts with and without ectopic WT-NADK2 expression.

C. Proline, glutamate and glutamine levels in the $NADK2^{mut}$ and $NADK2^{mut}$+WT fibroblasts. Data are the mean and SD from triplicates. ***$p < 0.001$, **$p < 0.01$ and *$p < 0.05$, calculated by unpaired two-tailed t test.

D. Proline, glutamate and glutamine levels in spent medium from the $NADK2^{mut}$ and $NADK2^{mut}$+WT fibroblasts. Data are the mean and SD from triplicates. ****$p < 0.0001$ and **$p < 0.01$, calculated by unpaired two-tailed t test.

medRxiv preprint doi: https://doi.org/10.64898/2026.10.28.26366400; this version posted October 29, 2026. The copyright holder for this preprint (which was not certified by peer review) is the author/funder, who has granted medRxiv a license to display the preprint in perpetuity.
It is made available under a CC-BY 4.0 International license .

E. Fractional enrichment of proline m+5 isotopologues in the NADK2^{mut} fibroblasts with or without WT-NADK2 expression. The cells were cultured in [U-^{13}C]glutamine for 6 hours. Data are the mean and SD from three replicates. ****p < 0.0001, calculated by unpaired two tail t test.

F. Quantitative RT-PCR analysis of *SLC38A1* and *SLC38A2* expression in NADK2^{mut} and NADK2^{mut}+WT fibroblasts.

Figure S6. Related to Figure 6. Analysis of *FDXR* variants and associated metabolic alterations in P3-0060 patient fibroblasts.

A. Evolutionary conservation of FDXR F51 and G437.

B. Heatmap showing the top-ranked altered metabolites in P3-0060 fibroblasts, and the effect of ectopic WT-FDXR on these metabolites.

medRxiv preprint doi: https://doi.org/10.64898/2026.10.28.26366400; this version posted October 29, 2026. The copyright holder for this preprint (which was not certified by peer review) is the author/funder, who has granted medRxiv a license to display the preprint in perpetuity.
It is made available under a CC-BY 4.0 International license .

Figure S1

A

Group	US cohort			Pakistan (PK) cohort		
	Sex	Age range	Subject #	Sex	Age range	Subject #
**Proband (=<18yr)**	F	0-18	**87**	F	0-18	**25**
	M	0-18	**88**	M	0-18	**33**
**Proband (>18yr)**	F	19-70	**16**	F	19-29	**1**
	M	19-70	**5**	M	19-29	**2**
**Sibling (=<18yr)**	F	0-18	**9**	F	0-18	**6**
	M	0-18	**6**	M	0-18	**5**
**Sibling (>18yr)**	F	19-29	**1**	F	-	**0**
	M	-	**0**	M	19-29	**2**
**Parent**	F	19-64	**42**	F	19-70	**58**
	M	19-90	**32**	M	19-60	**54**
**Grandparent**	F	50-70	**2**	F	-	**0**
	M	-	**0**	M	-	**0**
**Total**			**288**			**186**

B

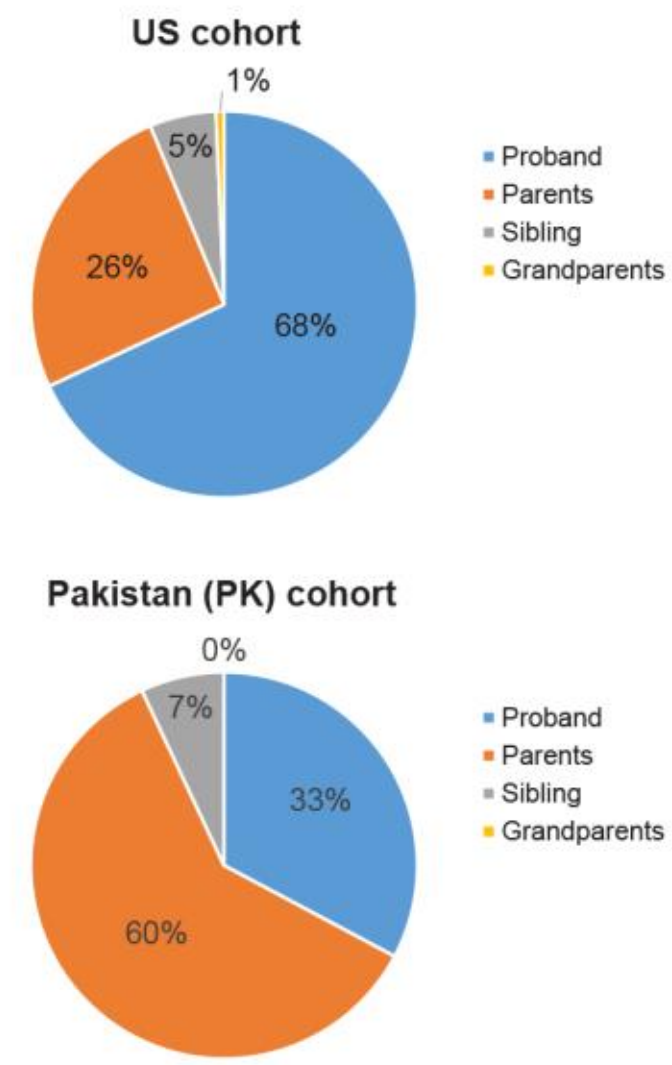

C

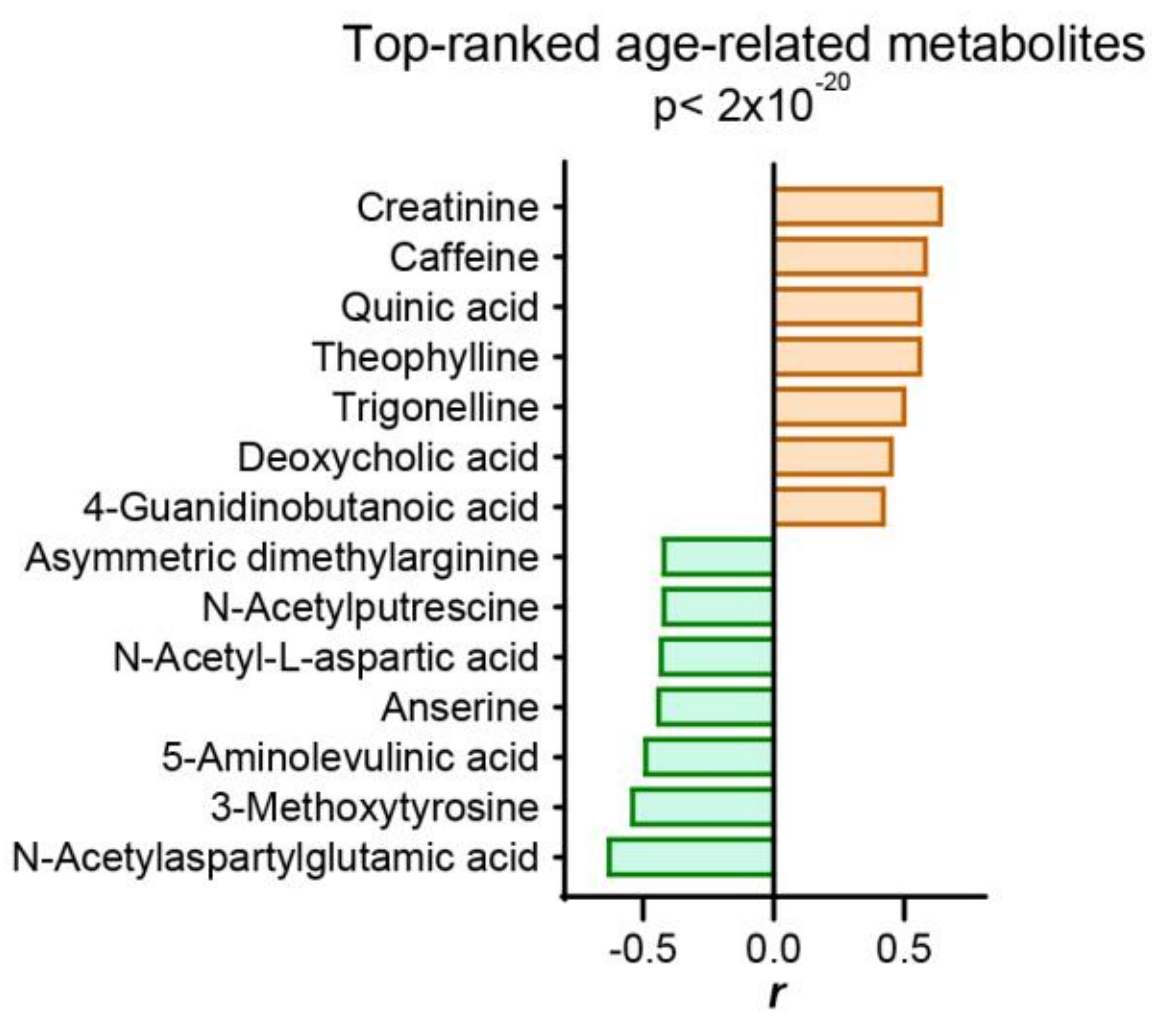

D

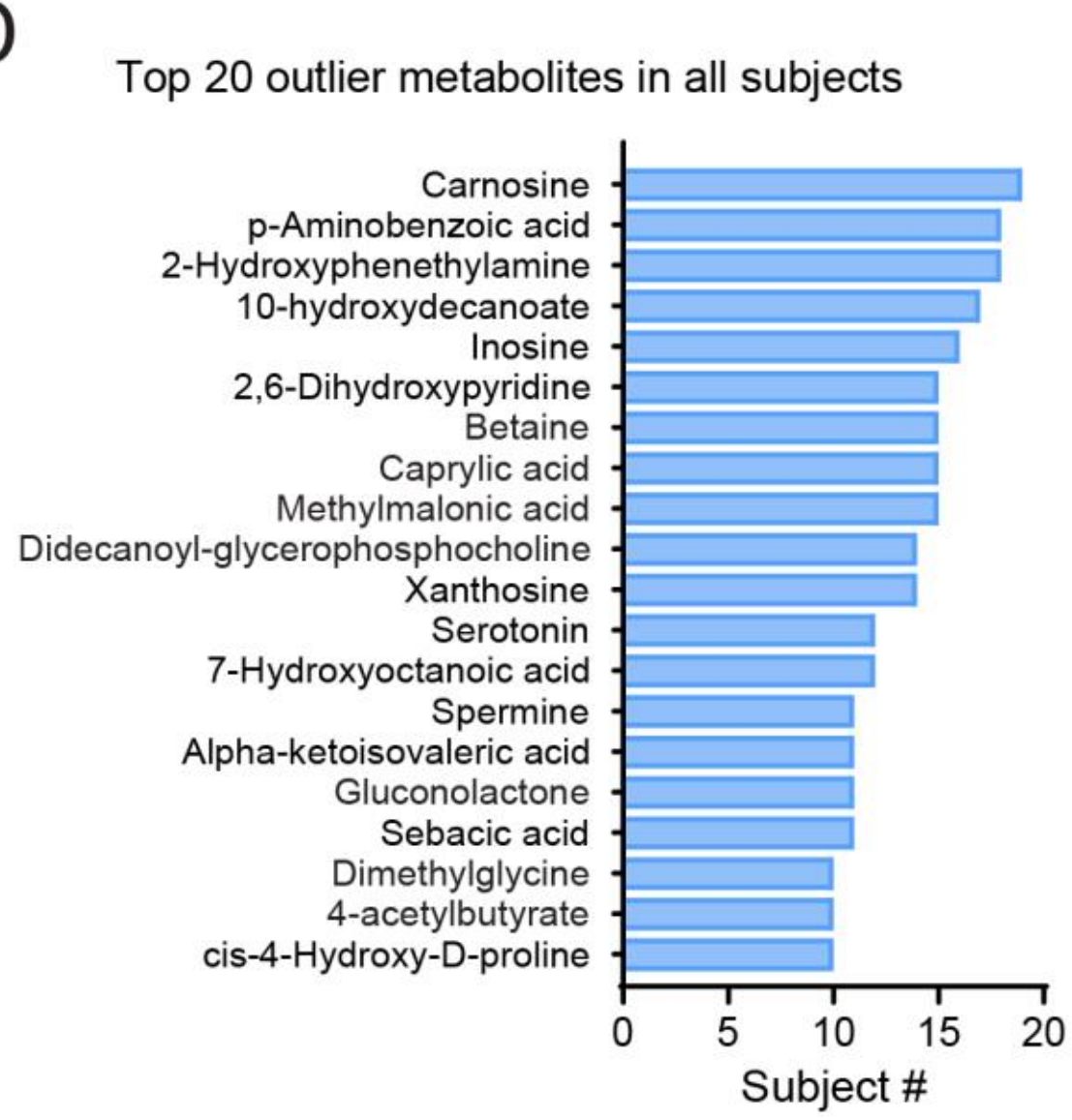

E

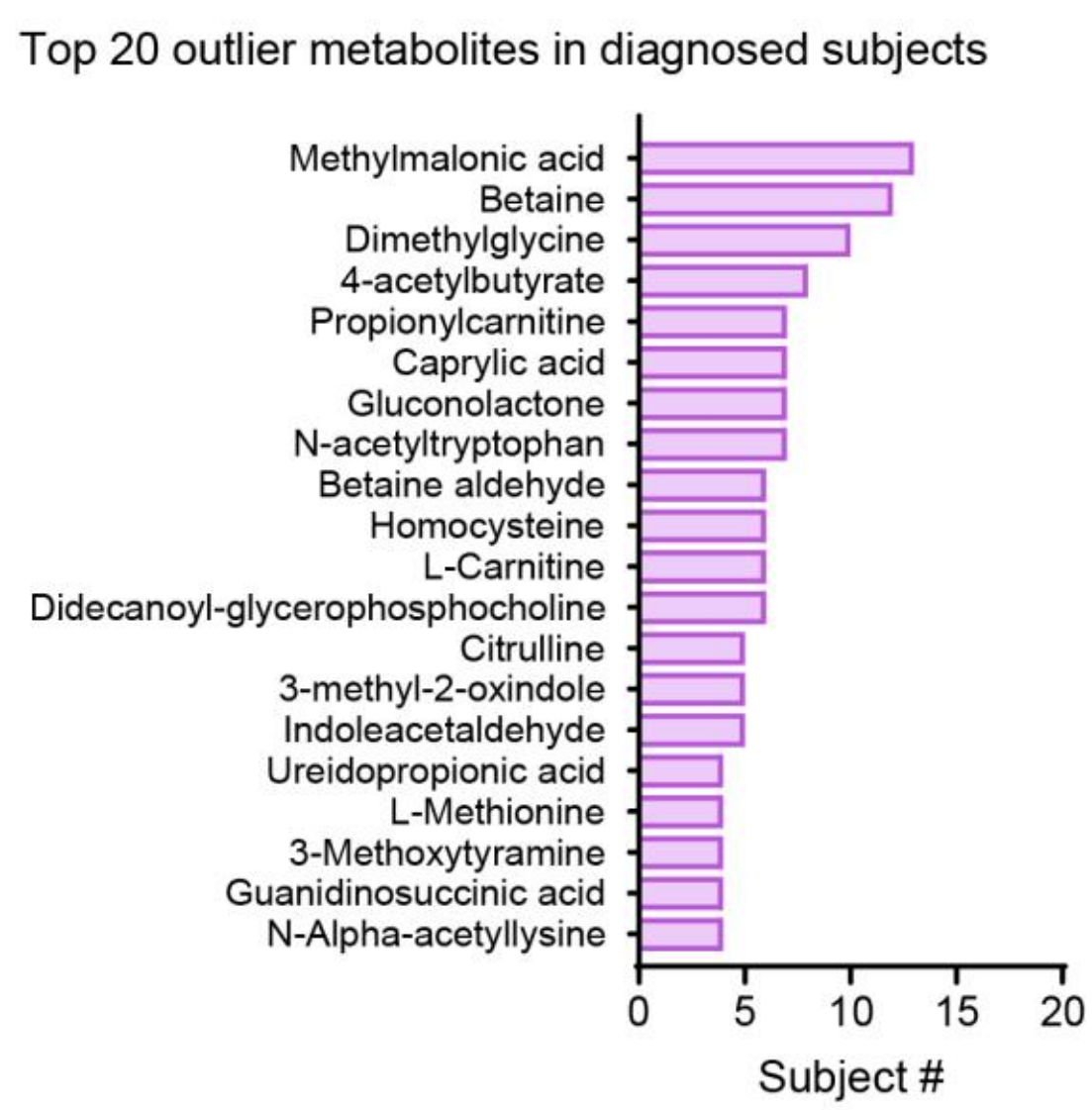

medRxiv preprint doi: https://doi.org/10.64898/2026.10.28.26366400; this version posted October 29, 2026. The copyright holder for this preprint (which was not certified by peer review) is the author/funder, who has granted medRxiv a license to display the preprint in perpetuity.
It is made available under a CC-BY 4.0 International license .

Figure S2

A

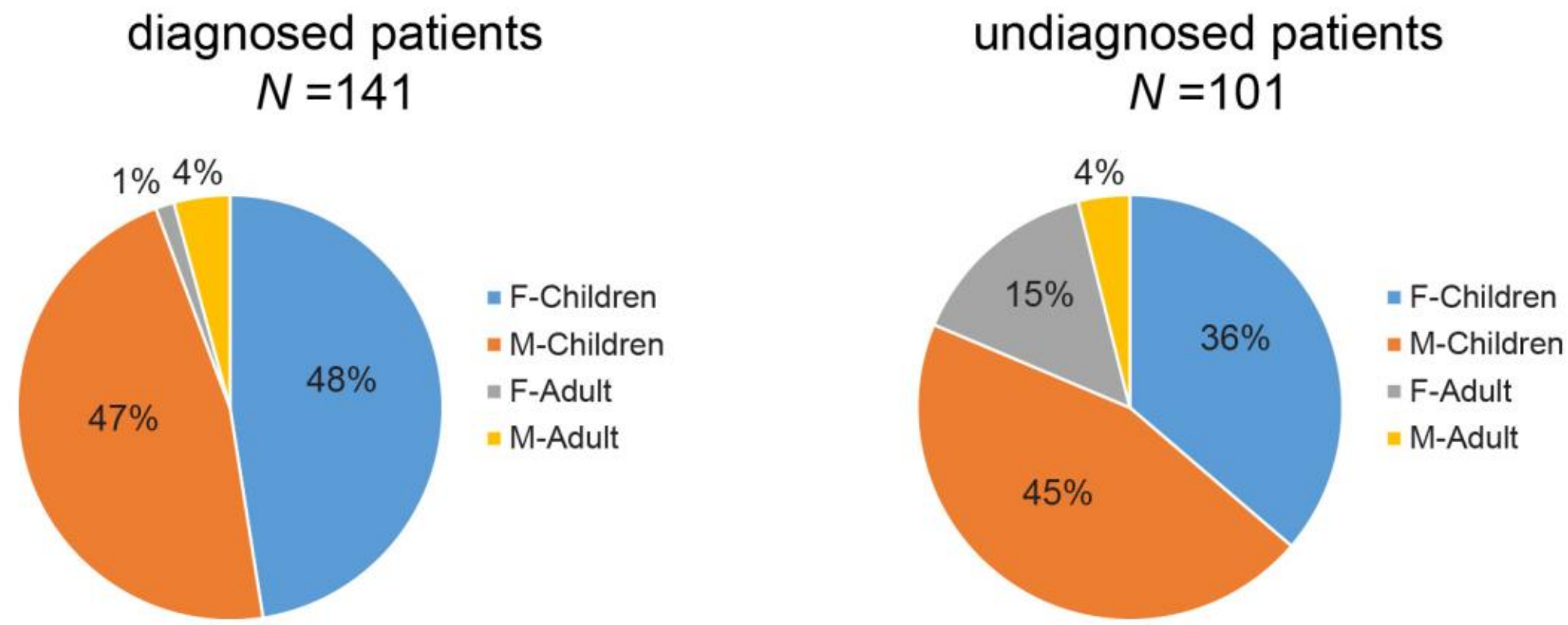

B

Metabolic pathway	Subject number
Amino acid	40
Carbohydrate	4
Cholesterol	4
Cofactor Vitamins	47
Energy metabolism	14
Lipid Fatty acid	5
Metabolite transport	13
Protein modification	8
Other	6
unknown	101
Total	***242***

C

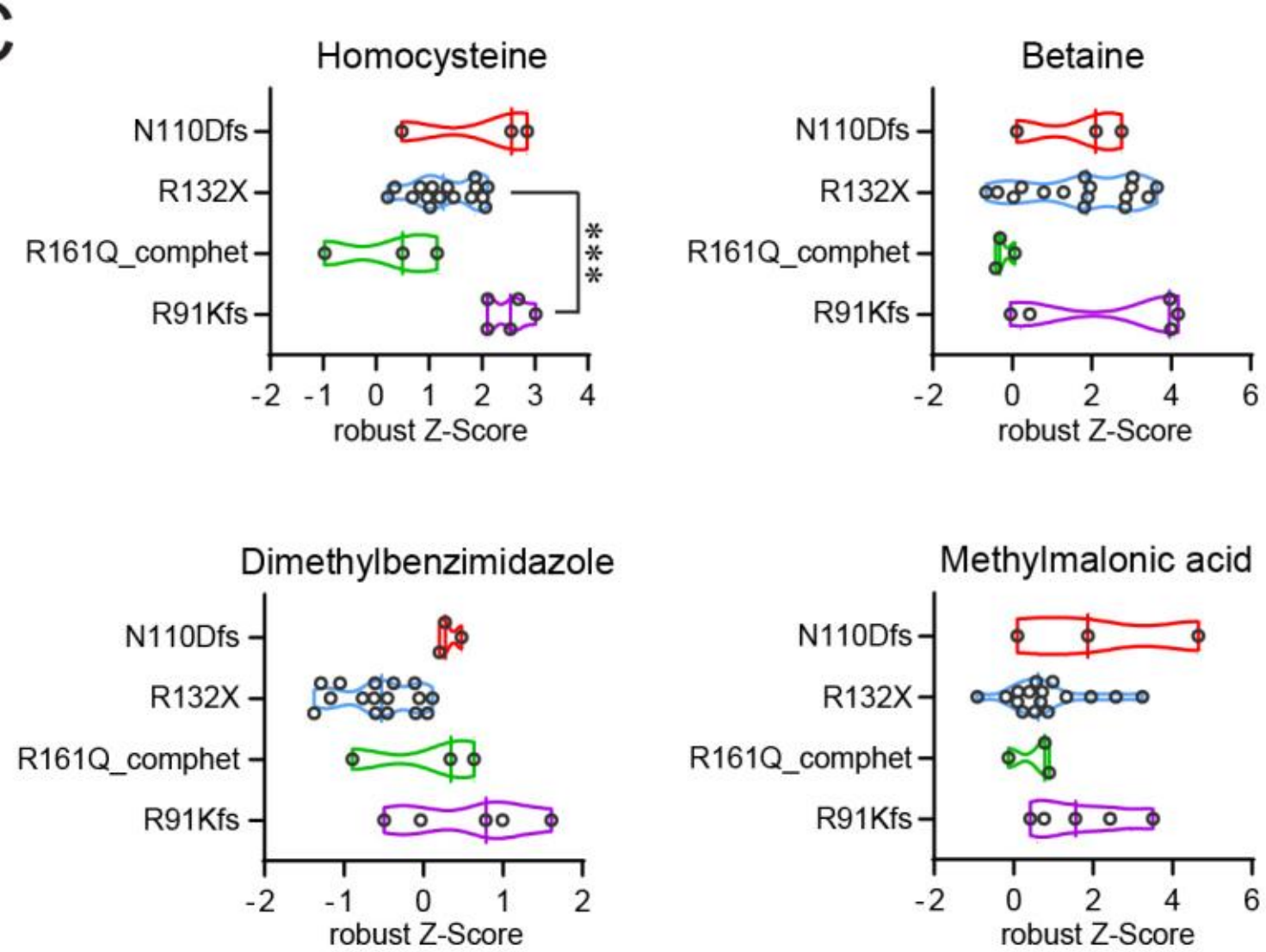

medRxiv preprint doi: https://doi.org/10.64898/2026.10.28.26366400; this version posted October 29, 2026. The copyright holder for this preprint (which was not certified by peer review) is the author/funder, who has granted medRxiv a license to display the preprint in perpetuity.
It is made available under a CC-BY 4.0 International license .

Figure S3

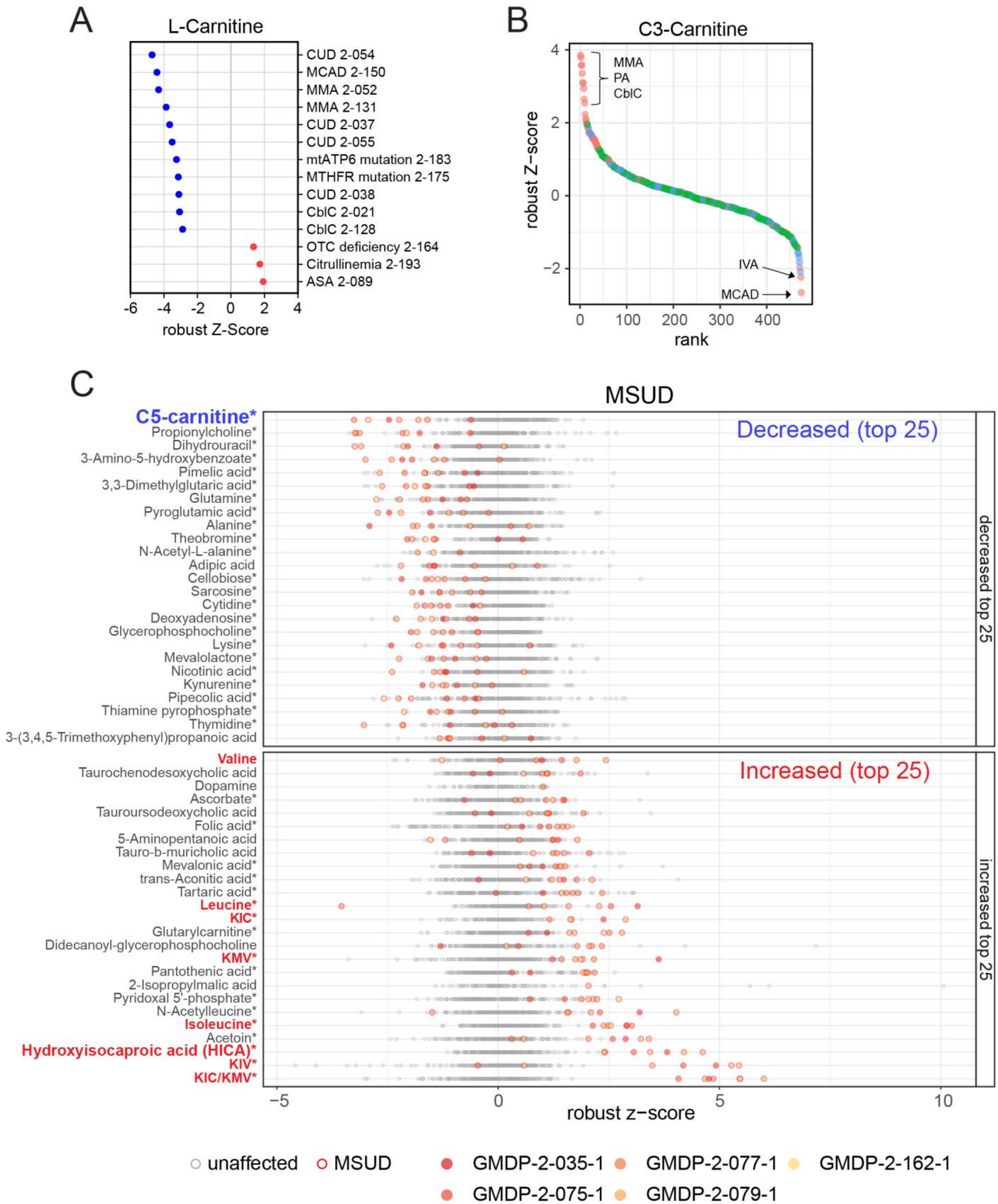

medRxiv preprint doi: https://doi.org/10.64898/2026.10.28.26366400; this version posted October 29, 2026. The copyright holder for this preprint (which was not certified by peer review) is the author/funder, who has granted medRxiv a license to display the preprint in perpetuity.
It is made available under a CC-BY 4.0 International license .

Figure S4

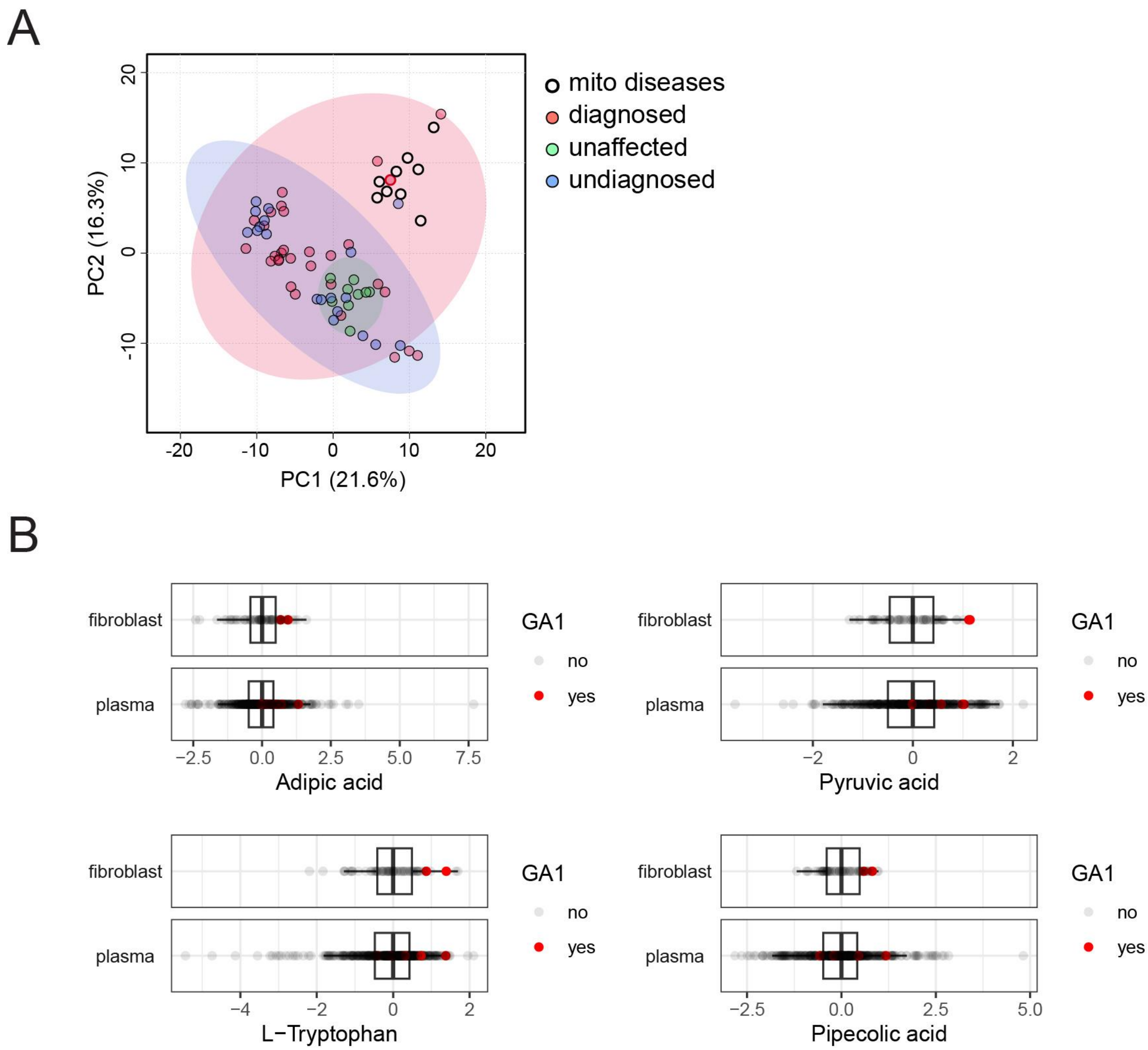

medRxiv preprint doi: https://doi.org/10.64898/2026.10.28.26366400; this version posted October 29, 2026. The copyright holder for this preprint (which was not certified by peer review) is the author/funder, who has granted medRxiv a license to display the preprint in perpetuity.
It is made available under a CC-BY 4.0 International license .

Figure S5

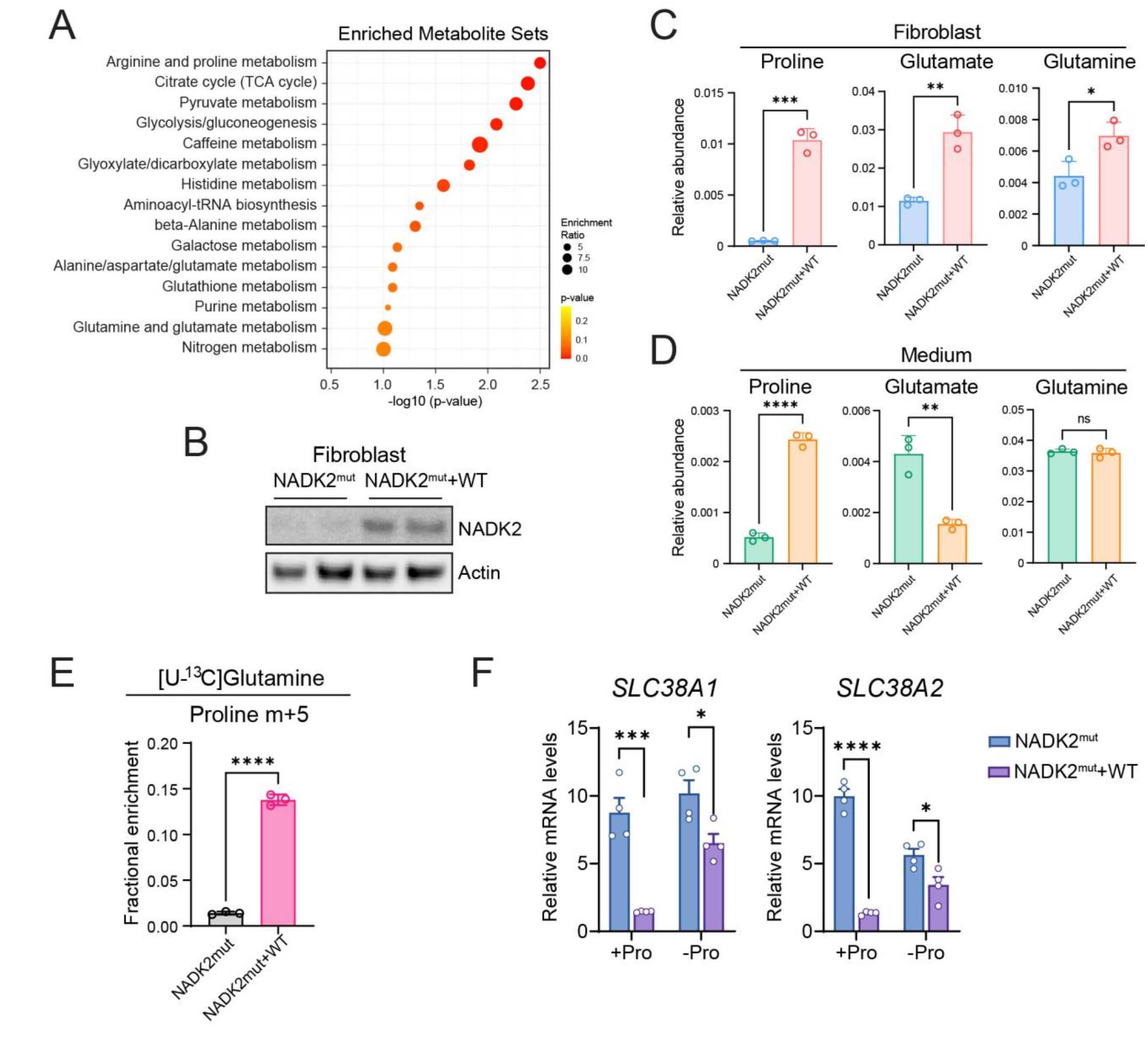

medRxiv preprint doi: https://doi.org/10.64898/2026.10.28.26366400; this version posted October 29, 2026. The copyright holder for this preprint (which was not certified by peer review) is the author/funder, who has granted medRxiv a license to display the preprint in perpetuity.
It is made available under a CC-BY 4.0 International license .

Figure S6

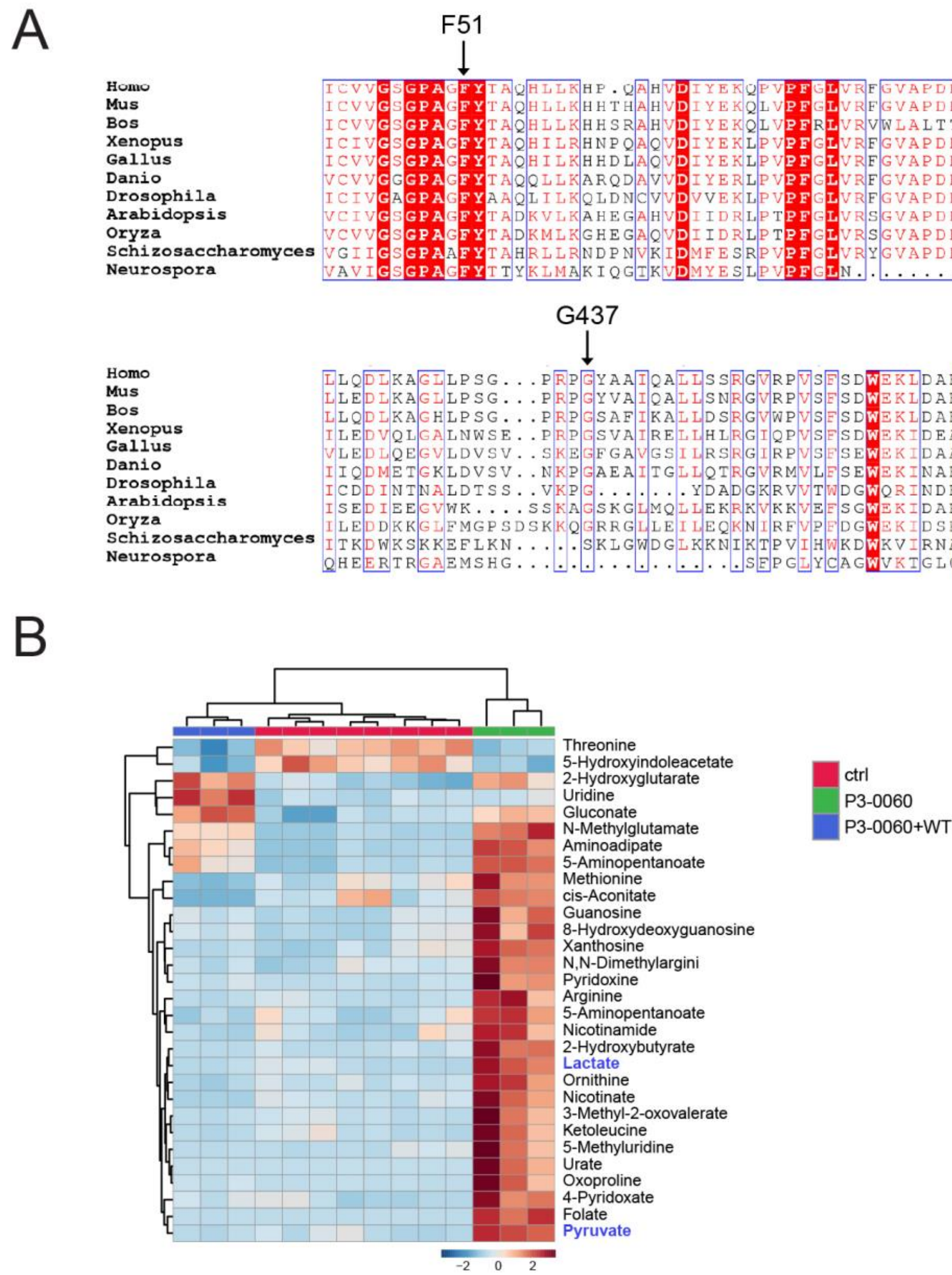

Supplement: Supplementary file 1 [file NIHPP2026.10.28.26366400v1-supplement-4.pdf]
